# Near Net Shape Manufacturing of Sheets from Al-Cu-Li-Mg-Sc-Zr Alloy

**DOI:** 10.3390/ma17030644

**Published:** 2024-01-28

**Authors:** Barbora Kihoulou, Rostislav Králík, Lucia Bajtošová, Olexandr Grydin, Mykhailo Stolbchenko, Mirko Schaper, Miroslav Cieslar

**Affiliations:** 1 Faculty of Mathematics and Physics, Charles University, Ke Karlovu 5, 121 16 Prague, Czech Republic; barbora.krivska@matfyz.cuni.cz (B.K.); rostislav.kralik@matfyz.cuni.cz (R.K.); lucibajtos@gmail.com (L.B.); 2 Chair of Materials Science, Paderborn University, Warburger Str. 100, 33098 Paderborn, Germany; grydin@lwk.upb.de (O.G.); stolbchenko@lwk.upb.de (M.S.); schaper@lwk.upb.de (M.S.)

**Keywords:** Al-Cu-Li-Mg-Zr alloys, Sc addition, twin-roll casting, constrained groove pressing, solution treatment, aging, pre-deformation, precipitation

## Abstract

Thin twin-roll cast strips from a model Al-Cu-Mg-Li-Zr alloy with a small addition of Sc were prepared. A combination of a fast solidification rate and a favorable effect of Sc microalloying refines the grain size and the size of primary phase particles and reduces eutectic cell dimensions to 10–15 μm. Long-term homogenization annealings used in conventionally cast materials lasting several tens of hours followed by a necessary dimension reduction through rolling/extruding could be substituted by energy and material-saving procedure. It consists of two-step short annealings at 300 °C/30 min and 450 °C/30 min, followed by the refinement and hardening of the structure using constrained groove pressing. A dense dispersion of 10–20 nm spherical ${\mathrm{Al}}_{3}\left(\mathrm{Sc},\mathrm{Zr}\right)$ precipitates intensively forms during this treatment and effectively stabilizes the structure and inhibits the grain growth during subsequent solution treatment at 530 °C/30 min. Small (3%) pre-straining after quenching assures more uniform precipitation of strengthening ${\mathrm{Al}}_{2}\mathrm{Cu}$ ($\theta^{\prime }$), ${\mathrm{Al}}_{2}\mathrm{CuMg}$ (S′), and Al_2_CuLi ($T_{1}$) particles during subsequent age-hardening annealing at 180 °C/14 h. The material does not contain a directional and anisotropic structure unavoidable in rolled or extruded sheets. The proposed procedure thus represents a model near net shape processing strategy for manufacturing lightweight high-strength sheets for cryogenic applications in aeronautics.

## 1. Introduction

Aluminum-lithium-copper-based alloys are often used in the space and aerospace industries. Compared to the conventional 2XXX and 7XXX series of aluminum alloys, they possess lower density and higher elastic modulus. Moreover, these alloys proved suitable for cryogenic application in several space-flight programs because of their high resistance to hydrogen-induced cracking [1,2,3,4].

The second generation of Al-Li-X alloys prepared by vacuum induction melting (VIM) suffers from several negative performance attributes associated with high crystallographic textures, strain localization, and anisotropy of mechanical properties. Their reduced ductility is generally attributed to the localization of stresses near shearable metastable ${\mathrm{Al}}_{3}\mathrm{Li}$ precipitates of ordered $\delta^{\prime }$-phase [5] or due to the presence of coarse particles of the stable phase Al_2_CuLi ($T_{1}/T_{2}$), decorating almost continuously grain boundaries [6,7]. Therefore, the third-generation alloys contain a higher Cu/Li ratio (the amount of Li does not exceed 2 wt.%), suppressing the formation of the deteriorating $\delta^{\prime }$-phase and substituting it with different strengthening phases, e.g., ${\mathrm{Al}}_{2}\mathrm{Cu}$ ($\theta^{\prime }$), ${\mathrm{Al}}_{2}\mathrm{CuMg}$ (S′), Al_2_CuLi ($T_{1}$), or a complex cubic phase ${\mathrm{Al}}_{5}{\mathrm{Cu}}_{6}{\mathrm{Li}}_{2}$ (σ) [8,9,10]. However, the anisotropy of mechanical properties still persists as a weakness due to intensive hot working (rolling, forging, extrusion) imposing unfavorable directional and textured structures. Polmear et al. [11] reported that a fine recrystallized grain structure exhibits almost isotropic tensile properties in the peak-aged condition even in the previous generation of Al-Li alloys. Applying severe plastic deformation (SPD) could lead to such grain refinement and directly influence precipitation kinetics [12]. Still, various negative phenomena might arise:

1. Precipitates/particles present before SPD can be fragmented, (partially) dissolved, or their growth and coarsening could occur, depending on temperature and strain rate [13,14,15,16,17].
2. SPD accelerates precipitation [13,18]; in many cases, the desired metastable phases are skipped, and the equilibrium phase is formed at a lower annealing temperature, often in the vicinity of numerous grain boundaries [15,17].

Recently, techniques such as equal channel angular pressing [19,20], high-pressure torsion [21], accumulative roll bonding [22,23], repetitive corrugation and strengthening [24], and constrained groove pressing (CGP) [25] have been used to produce ultrafine-grained (UFG) materials. They were also successfully used to process high alloyed Al-Cu-Li materials [13,26,27,28,29,30,31,32].

The temperature stability of the UFG microstructure could be vastly improved by adding Sc. This addition to Al-Cu-Li-Zr alloy activates the formation of stabilizing ${\mathrm{Al}}_{3}\left(\mathrm{Sc},\mathrm{Zr}\right)$ precipitates, impeding grain growth and shifting grain-coarsening to higher temperatures [33] so that the microstructure induced by SPD can withstand the necessary solution treatment at temperatures close to or above 500 °C.

A high density of grain boundaries and a lack of dislocations in the solution-treated materials are responsible for the preferential precipitation of coarse particles on the grain boundaries, leading to a depletion of the global distribution of strengthening phases in the matrix, limiting the performance/utilization of the alloy. Therefore, the T8 temper, which includes pre-deformation prior to aging, seems indispensable. Pre-deformation introduces dislocations as nucleation sites for strengthening phases, thus improving their homogeneous distribution in the matrix [34,35,36]. Pre-deformation in Al-Cu-Li alloys was reported to enhance strength, particularly due to the higher density of refined T1 precipitates [37,38,39,40].

Conventionally cast Al-Li-based materials face several significant issues originating in the scale of boundary primary phase particles requiring long-term homogenization treatment at high temperatures as a first post-processing step. Generally, temperatures close to 530 °C and soaking times longer than 10 h are required to dissolve or transform the primary phase particles and to receive a homogeneous distribution of main alloying elements [41]. Such long-term exposure to high temperatures is always coupled with a depletion of the ingot surface from Li atoms, and the scalping of ingots should always follow this annealing step. However, in the case of Sc-containing alloys, this long-term exposure to high temperatures results in a partial coarsening of Al_3_Sc precipitates or a formation of coarse AlCuSc particles [42,43,44]. It could significantly suppress the beneficial effect of Sc microalloying even in Sc and Zr-containing alloys with core-shell ${\mathrm{Al}}_{3}\left(\mathrm{Sc},\mathrm{Zr}\right)$ particles that are less sensitive to coarsening [45,46].

Recently, in addition to established Al-Li metal sheet processing based on VIM and subsequent cutting/rolling, twin-roll casting (TRC) can be applied to cast high alloyed materials [47,48]. High cooling rates (∼10^3^ K/s) received during TRC, and the possibility to cast strips or sheets almost at final gauges, yield several benefits. Except for energy and materials savings, grains formed during the solidification of TRC strips are usually smaller. Also, the dendritic structure formed during TRC is finer with tiny intermetallic particles of primary phases requiring less intensive exposure to high temperatures, preventing the undesirable Li-evaporation and the irreversible coarsening of ${\mathrm{Al}}_{3}\left(\mathrm{Sc},\mathrm{Zr}\right)$ precipitates—a typical feature of post-processed direct-chill (DC) or VIM materials [49,50].

The main concern of the present study is to show the peculiarities of Al-Cu-Li-Mg-Sc-Zr strips prepared by TRC and the potential of new post-processing avoiding energy-demanding and material-degrading homogenization treatment. A distribution of precipitates and total hardening were monitored in mold and twin-roll cast Sc-containing and Sc-free Al-Cu-Li-Mg-Zr strips subjected to one cycle of CGP without previous homogenization treatment. A beneficial effect of pre-deformation on aging response was demonstrated by investigating two aging tempers—T8 (solution heat treatment, pre-straining, artificial aging) and T6 (solution heat treatment, artificial aging).

## 2. Materials and Methods

### 2.1. Materials

Al-Cu-Li-Mg-Zr(-Sc) alloys were supplied in as-cast conditions—twin-roll cast and mold cast. Details of the preparation of TRC materials can be found in [48]. The preparation of aluminum sheets processed from DC cast or VIM ingots was represented by laboratory mold casting (MC). MC was performed under an argon protective atmosphere to an air-cooled graphite mold of 110 × 56 × 26 mm^3^. Irregularities and surface impurities of ingots were scalped to obtain a final block 85 × 50 × 22 mm^3^, which was used for further processing and study. The chemical composition of both alloys received from optical emission spectrometry (Q4 TASMAN) as an average value measured in three different positions of each material (TRC and MC) are given in Table 1. The main difference lies in the content of scandium: the Sc-free alloy will be denoted as AlLi, and the Sc-containing one as AlLiSc.

Since the CGP processing requires the material in the form of plates or sheets of limited dimensions, the MC ingots were sliced into rectangular strips of size 70 × 30 × 3 mm^3^. The TRC strips were cut into samples of similar dimensions, with the thickness determined by the gap between the rolls (∼3 mm) of the twin-roll caster. CGP comprises pressing in asymmetrically positioned grooves and straightening between two flat dies. Groove die geometry (described in [51]) generates alternating deformed and undeformed regions after one corrugation and straightening step. Due to the asymmetry of the groove die, a rotation of the sample by 180° along the axis perpendicular to the plane of the sample allows for the deformation of the undeformed region during the next corrugation and straightening step. These four pressings are considered as one CGP cycle resulting in a homogeneous effective strain of 1.16 throughout the sample [51].

All materials were CGP deformed using one complete cycle. In TRC alloys, the grooves of dies were oriented parallel to the rolling direction (RD). Before CGP, the materials were subjected to two-step annealing at 300 °C/30 min and 450 °C/30 min, finished by quenching in water at room temperature (RT) after each annealing. This treatment aims to obtain a fine dispersion of ${\mathrm{Al}}_{3}\left(\mathrm{Sc},\mathrm{Zr}\right)$ (Sc-containing alloys) or Al_3_Zr (Sc-free alloys) precipitates hindering/decelerating recrystallization and grain growth.

Before the CGP deformation, the dies and samples were preheated at 300 °C. The preheating of the samples lasted 5 min. The temperature of the dies was held at 300 °C during the entire process of deformation. Processed materials were then air-cooled to RT.

After CGP, the materials were subjected to the solution treatment at 530 °C/30 min with water quenching and then aged at 180 °C for up to 110 h (T6 temper) or pre-strained (3%) before the final aging (T8 temper). All annealings above 180 °C were performed in an air resistance furnace, while age hardening at 180 °C was performed in a thermostat with a silicon oil bath. Pre-deformation was carried out using a universal testing machine INSTRON 5882 at a strain rate of $5\times 10^{-4}\;s^{-1}$ to reach 3% plastic deformation in compression. The dimensions of the samples were 20 mm × 5 mm × original sheet thickness. The compression was performed in the direction of grooves (in RD in the case of TRC materials). The same direction was chosen for microhardness indentation.

### 2.2. Methods

Precipitation strengthening during artificial aging was monitored using the Vickers microhardness method with a fully automated hardness tester Qness Q10. A 100 g load and a dwell time of 10 s were used in the experiment. At least 50 indentations were performed to calculate the average value of the microhardness of each sample.

Microstructural observations were performed by light optical (LOM), scanning electron (SEM), and transmission electron microscopes (TEM). Samples for metallography and SEM were mechanically ground on SiC papers and subsequently polished by diamond suspensions and the Struers OPS colloidal silica suspension. A cross-sectional view of the material structure (grains/secondary phase particle distribution) was acquired by a LOM Zeiss Axio Observer (Carl Zeiss AG, Jena, Germany). Observations in the polarized light revealed a grain structure for which the polished sample surface was anodized with Barker’s reagent in Lectropol 5 at 10 °C. The distribution of primary/secondary phase particles and grain orientation was investigated by a scanning electron microscope FEI Quanta 200F (Thermo Fisher Scientific Inc., Waltham, MA, USA) equipped with the energy dispersive spectroscopy detector and the detector of electron backscatter diffraction (EBSD). To collect grain orientation maps using EBSD, the sample surface was electropolished by a 30% solution of HNO_3_ in methanol after regular mechanical grinding and polishing. EBSD quick grain maps processed in OIM analysis software were used for grain size evaluation according to the standard line intercept method. The relation for the grain size *d* is given by

(1) $$d=k\cdot \frac{\pi D}{n},$$

where *D* is a testing circle diameter and *n* is the number of intersected grains [52]. The added factor *k* is a proportionality constant between an average intercept length, determined from a 2D section (e.g., experimental micrograph), and the “real” 3D grain size [53,54]. The *k* value depends on the geometry and for nontextured grains of terakaidecahedral shape was estimated to be k≐1.56 [53]. The circle intercept method was applied three times per micrograph and the average with the standard deviation of the measurement is shown in Section 3.

TEM analyses were performed on 3 mm diameter discs, which were mechanically thinned on SiC papers and subsequently twin-jet electropolished (Tenupol 5), using the solution of HNO_3_ in methanol cooled to −20 °C. All observations were performed in the direction perpendicular to the grooves. A JEOL JEM 2200FS (JEOL, Tokyo, Japan) electron microscope operated at 200 kV was used for TEM observations. The microscope is equipped with a JEOL Centurio large-angle EDS detector (JEOL, Tokyo, Japan).

## 3. Results

### 3.1. Microstructure Studies of the As-Cast Materials

**Figure 1:**
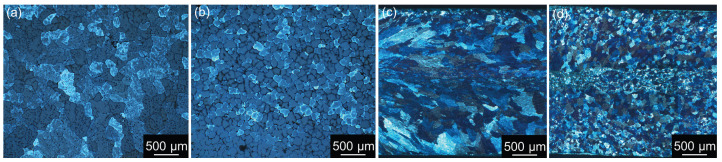
LOM images of the as-cast materials showing grain distributions in (**a**) AlLi MC, (**b**) AlLiSc MC, (**c**) AlLi TRC, and (**d**) AlLiSc TRC.

Figure 1 shows typical distributions of grains in all as-cast materials. Mold-cast materials contain almost equiaxed grains with nearly uniform grain size (Figure 1a,b). In the case of twin-roll cast material, a significantly larger surface-to-bulk region ratio led to a somewhat uneven grain size through the strip thickness: small and equiaxed grains are typical for surface and central parts. In contrast, coarser grains inclined in the rolling direction are in bands surrounding the center of the strip (Figure 1c,d). Such a structure is characteristic of twin-roll cast Al alloys, which results from faster cooling and induced deformation by rolling [48]. The role of Sc addition is demonstrated by a reduction in the grain size in both MC and TRC materials. Compared with the AlLi TRC strip, the grain size in the AlLiSc TRC alloy is more homogeneous, because the addition of Sc effectively prevents the formation of coarser grains in bands surrounding the central part.

**Figure 2:**
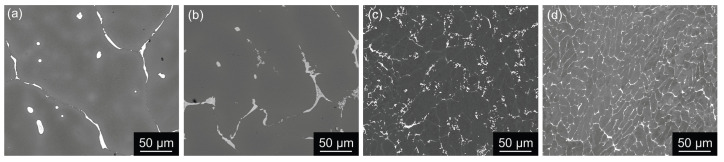
SEM BSE images of eutectic cells in as-cast materials: (**a**) AlLi MC, (**b**) AlLiSc MC, (**c**) AlLi TRC, and (**d**) AlLiSc TRC, demonstrating a positive influence of TRC and Sc addition on the refinement of the structure.

The cooling rate also significantly impacts the distribution of primary phases. Considerably coarser boundary Al-Cu and Al-Cu-Fe rich phases (Figure 2a,b) were observed in the MC materials compared to the TRC materials (Figure 2c,d). The eutectic cell size (interdendritic spacing) *L* was evaluated by the linear intercept method from a set of SEM images covering the area 1200×1200 μm^2^ in the MC materials and 250×250 μm^2^ in the TRC materials. The *L* values in Table 2 show the dominant role of the solidification rate. The cell size in both TRC materials is almost ten times smaller than in the MC materials. The Sc microalloying provides an easy-to-distinguish homogeneous distribution of primary phase particles in the cell boundaries of the TRC materials (Figure 2c,d).

**Figure 3:**
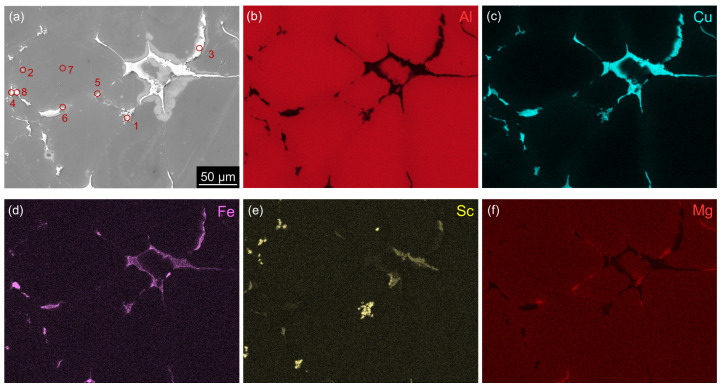
BSE image with corresponding EDS maps showing the distribution of Al, Cu, Fe, Sc, and Mg in a selected zone containing rare Sc-rich particles in the MC AlLiSc alloy in the as-cast state.

The chemical composition of primary phases was monitored by EDS mapping, disclosing a partially less homogeneous distribution of Sc in the Sc-containing MC material because coarser Sc-rich particles can sporadically be found in the MC alloy (Figure 3). No such particles were found in the TRC AlLiSc material. Only qualitative results in Table 3 do not allow for a direct determination of particular phases due to a strong influence of the surrounding matrix and particles overlapping. However, the primary phases containing the main alloying elements were identified by TEM in our previous studies, showing the presence of stable equilibrium phases ${\mathrm{Al}}_{2}\mathrm{Cu}$, ${\mathrm{Al}}_{2}\mathrm{CuLi}$, ${\mathrm{Al}}_{2}\mathrm{CuMg}$ [55], and ${\mathrm{Al}}_{7}{\mathrm{Cu}}_{2}\mathrm{Fe}$ [56].

**Figure 4:**
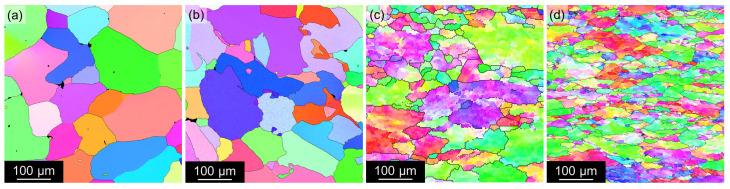
EBSD inverse pole figures of as-cast materials: (**a**) AlLi MC, (**b**) AlLiSc MC, (**c**) AlLi TRC, and (**d**) AlLiSc TRC. Central parts of strips were selected in the TRC materials.

Grain size and orientation were studied via EBSD mapping. Figure 4 shows a region of 500 × 500 $\mu m^{2}$ confirming the recrystallized structure in mold-cast materials independently of the presence of Sc. A significant fragmentation of grains into subgrains was found in TRC alloys reflecting the influence of small deformation imposed by rolls during the process of TRC.

**Figure 5:**
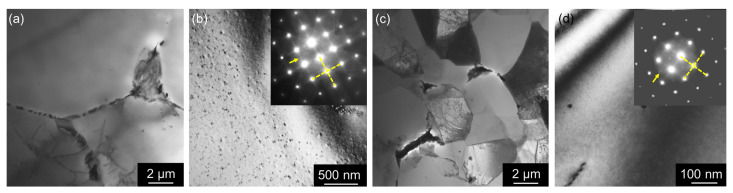
TEM micrographs of the as-cast AlLiSc materials: (**a**,**b**) MC, (**c**,**d**) TRC. Zone [001], both the yellow arrows in insets of diffraction patterns (**b**,**d**) pointing at the position of the superstructural spots and streaks highlighted by the yellow dashed lines reflect the presence of fine $\theta^{\prime }$-Al_2_Cu precipitates.

Figure 5 shows TEM images comparing MC and TRC AlLiSc alloys, where (a) and (c) display the (sub)grain structure with the location and size of coarse boundary phases, and (b) and (d) display the distribution of the $\theta^{\prime }$-Al_2_Cu precipitates with corresponding diffraction patterns in zone [001] in insets. Numerous Al_2_Cu precipitates form in the MC material (Figure 5b), while their presence in the TRC alloy is evidenced only by the diffraction pattern.

### 3.2. Microstructure Studies of CGP Materials

**Figure 6:**
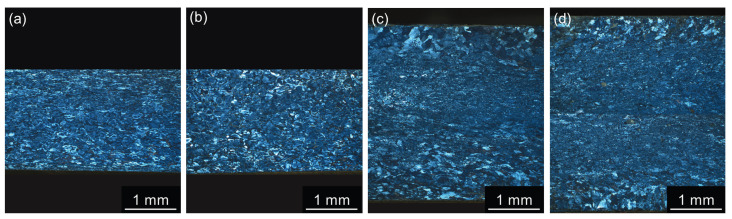
LOM images of materials after annealing at 300 °C/30 min, 450 °C/30 min, and one CGP cycle performed at 300 °C: (**a**) AlLi MC, (**b**) AlLiSc MC, (**c**) AlLi TRC, and (**d**) AlLiSc TRC.

The distribution of grains in samples after one CGP cycle is shown in Figure 6. Grain refinement and more uniform grain distribution are apparent in all materials compared to the as-cast structures displayed in Figure 1.

**Figure 7:**
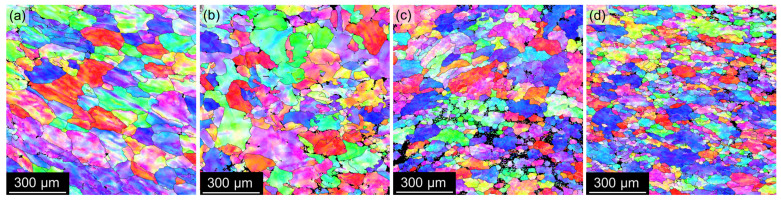
EBSD IPF maps of materials after annealing at 300 °C/30 min, 450 °C/30 min, and one CGP cycle performed at 300 °C: (**a**) AlLi MC, (**b**) AlLiSc MC, (**c**) AlLi TRC, and (**d**) AlLiSc TRC.

Moreover, EBSD analyses show further fragmentation of grains into numerous subgrains (see IPF maps in Figure 7 acquired in the central region of the strips). The black color in the IPF maps marks regions with a poor confidence index, which is caused by the presence of coarser non-α-Al phases or central segregations in the case of TRC materials.

**Figure 8:**
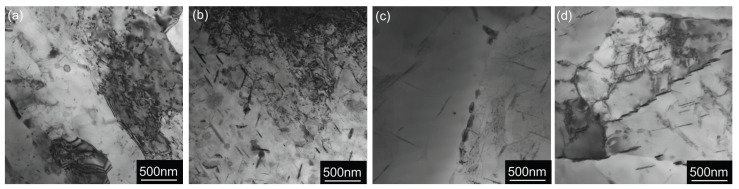
TEM micrographs showing the distribution of Cu and Mg-rich particles in materials after annealing at 300 °C/30 min, 450 °C/30 min, and one CGP cycle performed at 300 °C: (**a**) AlLi MC, (**b**) AlLiSc MC, (**c**) AlLi TRC, and (**d**) AlLiSc TRC.

TEM analysis of the samples after CGP processing (Figure 8) shows overaged Cu- and Mg-rich particles (identified similarly as in the as-cast state as Al_2_Cu-$\theta^{\prime }$ in the form of coarser plates and Al_2_CuMg-$S^{\prime }$ in the form of needles [48]).

### 3.3. Solution Treatment and Aging

**Figure 9:**
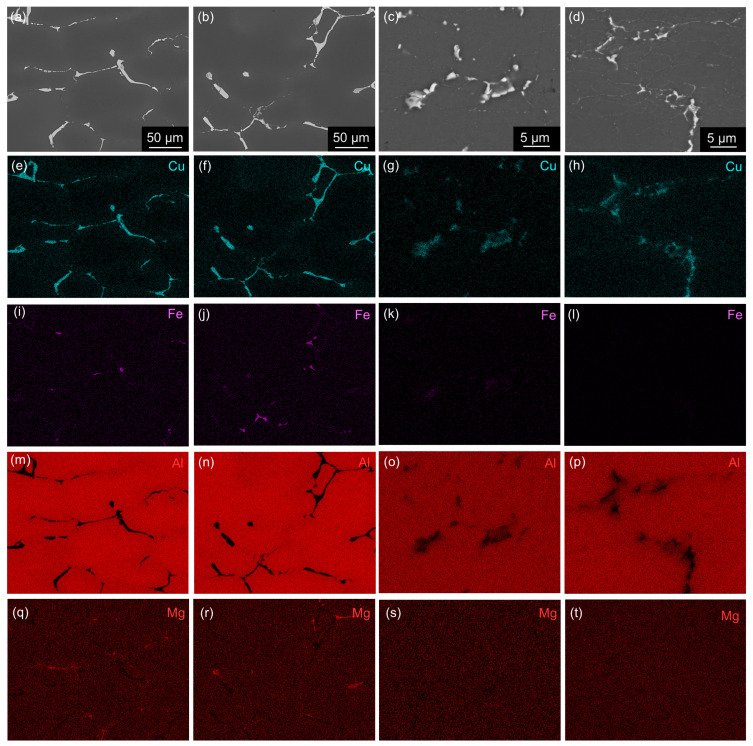
SEM BSE images and corresponding EDS maps of materials after annealing at 300 °C/30 min, 450 °C/30 min, and one CGP cycle performed at 300 °C: (**a**,**e**,**i**,**m**,**q**) AlLi MC, (**b**,**f**,**j**,**n**,**r**) AlLiSc MC, (**c**,**g**,**k**,**o**,**s**) AlLi TRC, and (**d**,**h**,**l**,**p**,**t**) AlLiSc TRC.

**Figure 10:**
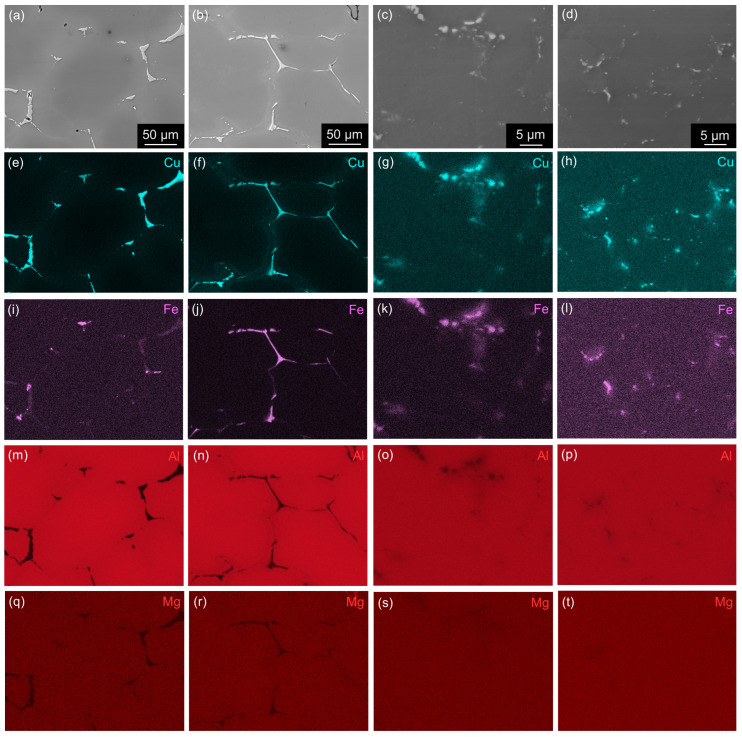
SEM BSE images and corresponding EDS maps of materials after annealing at 300 °C/30 min, 450 °C/30 min, one CGP cycle, and one solution treatment at 530 °C/30 min: (**a**,**e**,**i**,**m**,**q**) AlLi MC, (**b**,**f**,**j**,**n**,**r**) AlLiSc MC, (**c**,**g**,**k**,**o**,**s**) AlLi TRC, and (**d**,**h**,**l**,**p**,**t**) AlLiSc TRC.

All materials deformed by CGP were subjected to solution treatment and aging (T6 grade) or solution treatment, pre-straining (3%), and aging (T8 grade). Figure 9 and Figure 10 show the distribution of Al, Cu, Mg, and Fe before and after solution treatment (530 °C/30 min). Elemental mapping in TRC materials was performed under higher magnification due to the smaller size of primary phases. The most remarkable result of the EDS analysis is a complete dissolution of Mg-bearing particles and a majority of Cu-rich particles in the TRC materials. Only complex phases containing Fe and Cu remained undissolved. On the other hand, MC materials always contain Fe-free, coarser, undissolved Cu-rich particles even after this annealing. This model processing combines solution and homogenization treatments into one step but also clearly shows that the temperature/duration of this treatment was suitable for TRC alloys but insufficient for MC materials, where Al-Cu phases remained undissolved.

**Figure 11:**
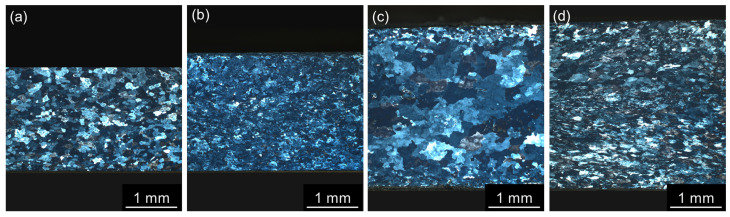
LOM images of materials after annealing at 300 °C/30 min, 450 °C/30 min, one CGP cycle, and one solution treatment at 530 °C/30 min: (**a**) AlLi MC, (**b**) AlLiSc MC, (**c**) AlLi TRC, and (**d**) AlLiSc TRC.

**Figure 12:**
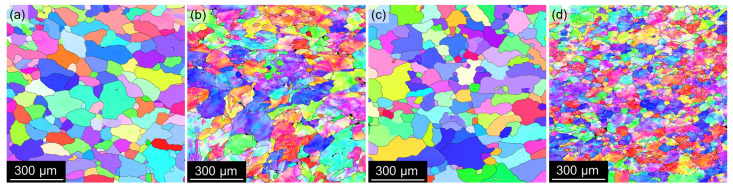
EBSD IPF maps of materials after annealing at 300 °C/30 min, 450 °C/30 min, one CGP cycle, and one solution treatment at 530 °C/30 min: (**a**) AlLi MC, (**b**) AlLiSc MC, (**c**) AlLi TRC, and (**d**) AlLiSc TRC.

As expected, Sc-containing alloys exhibit good resistance to grain coarsening during this solution treatment (Figure 11b,d), while recrystallization and grain growth occur in the Sc-free materials (Figure 11a,c). Moreover, EBSD analysis shows that the substructure is also preserved in the AlLiSc alloys (see IPF maps in Figure 12b,d). On the contrary, the IPF maps of the AlLi alloys show almost zero misorientation within individual grains (Figure 12a,c), confirming complete recrystallization. Table 4 compares grain sizes of all materials after the solution treatment. The positive role of Sc was confirmed in both the MC and the TRC materials. However, the finer microstructure imposed by CGP on the TRC material persists, and this material exhibits the finest grain size.

**Figure 13:**
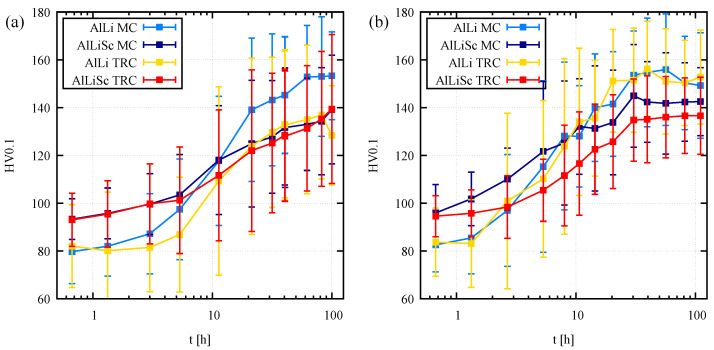
Microhardness evolution during aging: (**a**) specimen without pre-straining after the solution treatment, (**b**) specimen pre-strained by 3% after the solution treatment.

The precipitation strengthening was further studied by microhardness measurements on both non-pre-strained (Figure 13a) and pre-strained materials (Figure 13b) during aging at 180 °C. Values displayed graphically in Figure 13 are the averages calculated from ∼70 points measured through the whole strip thickness. The area used for indentation was set perpendicularly to the CGP die grooves to include a possible scatter of microhardness fluctuations caused by the periodicity of the groove dies.

The first point was measured after solution treatment (and pre-straining for the T8 temper) and subsequent aging at 180 °C/40 min, to minimize the influence of natural aging during the necessary manipulation. At this point, the MC and TRC Sc-free graphs fully overlap, just like those of MC and TRC alloys containing Sc. This separation (Sc-free or Sc-containing ones) could result from the grain size unification and the role of subgrains. Most probably, the main contribution influencing the initial microhardness does not include the precipitation strengthening by the anticipated main strengthening phases $\theta^{\prime }$, $T_{1}$, $S^{\prime }$, σ because the aging time is still noticeably short. A distinguishable increase in microhardness can be noticed after 5 h of the artificial aging in non-pre-strained materials (see Figure 13a). A significantly higher relative increase in microhardness could be noticed in Sc-free alloys. In pre-strained materials, the microhardness increases after 80 min of aging (see Figure 13b) and the peak microhardness was reached after shorter aging times than in non-pre-strained materials. Lower values of microhardness in the Sc-containing materials could be recognized.

**Figure 14:**
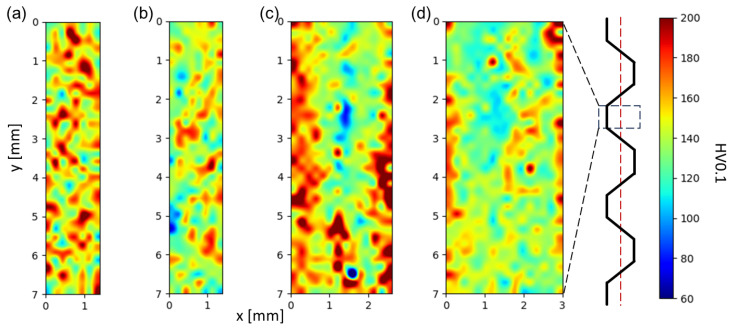
Distribution of microhardness through the sample thickness of materials after annealing at 300 °C/30 min, 450 °C/30 min, one CGP cycle performed at 300 °C, one solution treatment at 530 °C/30 min, 3% pre-straining and aging at 180 °C/110 h: (**a**) AlLi MC, (**b**) AlLiSc MC, (**c**) AlLi TRC, and (**d**) AlLiSc TRC.

However, the microhardness results should be interpreted with caution. The averaged microhardness values carry a large scatter. The standard deviation reached about 20 HV0.1 in all materials during the aging treatment. Within this variance, the microhardness curves are almost identical. However, several essential remarks follow from the evolution of the standard deviation. The scatter is maximal in the steep part of the microhardness curves, where they exhibit the highest strengthening rate. The scatter is more significant in materials without pre-straining (Figure 13a), and small pre-straining partially suppresses it. The mapping of microhardness after solution treatment, pre-straining, and aging reflects the features of the scatter (Figure 14). MC alloys contain coarse primary phases, which were not fully dissolved during the solution treatment, and larger microhardness values were reached near the remaining primary phases. By contrast, central segregations or enriched regions near the strip surface occasionally occurred in TRC alloys, leading to local microhardness variations.

**Figure 15:**
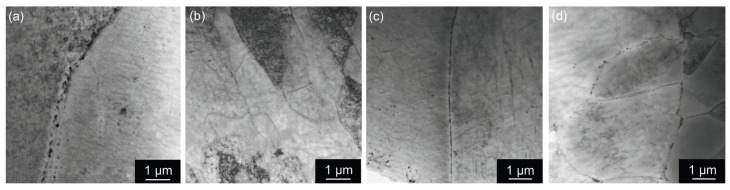
TEM micrograph of materials after annealing at 300 °C/30 min, 450 °C/30 min, one CGP cycle performed at 300 °C, one solution treatment at 530 °C/30 min, and aging at 180 °C/40 h: (**a**) AlLi MC, (**b**) AlLiSc MC, (**c**) AlLi TRC, and (**d**) AlLiSc TRC.

The scatter generally reflects the extent of inhomogeneity in the structure, amplified by the influence of strengthening particles. Such an inhomogeneous structure imposing non-uniform distribution of strengthening particles on (sub)grain boundaries was observed in TEM in materials without pre-straining (see Figure 15) after aging at 180 °C/40 h, representing the near peak age T6 condition—just before reaching the maximal microhardness.

**Figure 16:**
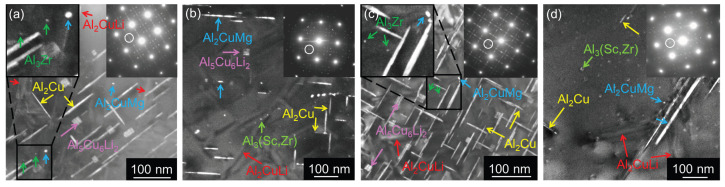
TEM micrograph of materials after annealing at 300 °C/30 min, 450 °C/30 min, one CGP cycle performed at 300 °C, one solution treatment at 530 °C/30 min, and aging at 180 °C/40 h (near peak age condition), dark field, zone [001]: (**a**) AlLi MC, (**b**) AlLiSc MC, (**c**) AlLi TRC, and (**d**) AlLiSc TRC. Small Al_3_Zr particles are highlighted in insets in (**a**,**c**).

**Figure 17:**
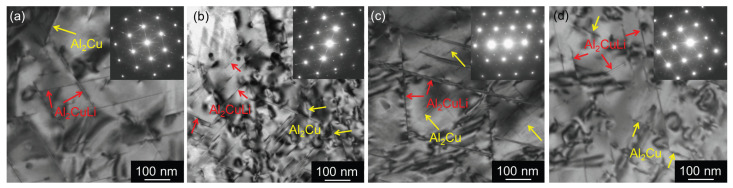
TEM micrograph of materials after annealing at 300 °C/30 min, 450 °C/30 min, one CGP cycle performed at 300 °C, one solution treatment at 530 °C/30 min, and aging at 180 °C/40 h (near peak age condition), zone [110]: (**a**) AlLi MC, (**b**) AlLiSc MC, (**c**) AlLi TRC, and (**d**) AlLiSc TRC.

**Figure 18:**
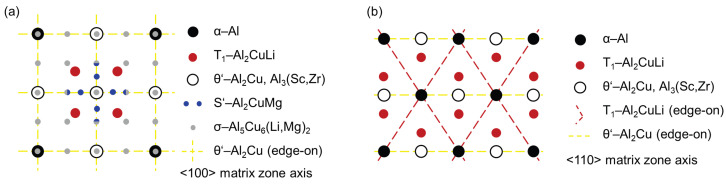
Schematic diagrams of the diffraction pattern along: (**a**) 〈100〉, (**b**) 〈110〉 zone axes.

**Figure 19:**
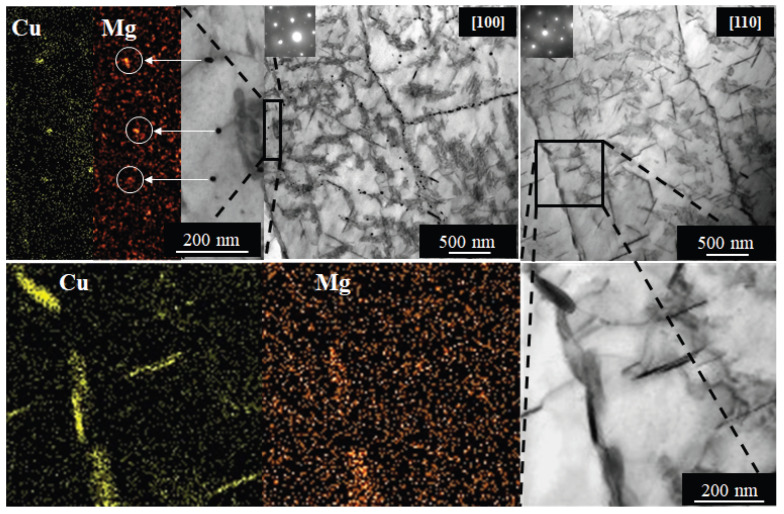
TEM images showing the same area in [100] and [110] orientations and corresponding EDS maps of needle-shaped subgrain boundary particles containing Cu and Mg. Arrows indicate Mg maps of selected particles highlighted by circles.

Strengthening $\theta^{\prime }$ plates (Al_2_Cu) in {100} planes, $T_{1}$ plates (Al_2_CuLi) in {111} planes, $S^{\prime }$ needles (Al_2_CuMg) elongated in <100> directions, σ cuboids (Al_5_Cu_6_(Li,Mg)), and spherical Al_3_(Zr,Sc) and Al_3_Zr particles were studied near zones [001] (Figure 16) and [110] (Figure 17). Selected area electron diffraction analysis based on schematical diffraction patterns shown in Figure 18 was combined with local EDS analysis and tilting experiments to identify particular precipitates. While small spherical Al_3_Zr (Figure 16a,c) and Al_3_(Sc,Zr) (Figure 16c,d) particles were formed already during the two-step annealing (300 °C/30 min + 450 °C/30 min) before CGP and further coarsened during solution annealing (530 °C/30 min), the remaining particles crystallized during artificial aging at 180 °C. Significantly sparser distributions of $\theta^{\prime }$ and $T_{1}$ precipitates in Al-Li-Sc alloys were observed in the orientation [100], particularly in the (sub)grain interiors (compare Figure 16a,c with Figure 16b,d). A closer analysis of coarse precipitates shows a prevailing formation of needle-shape $S^{\prime }$-Al_2_CuMg particles at the expense of $\theta^{\prime }$ and $T_{1}$ plates. Cu and Mg in those particles (Figure 19) further confirm this statement.

**Figure 20:**
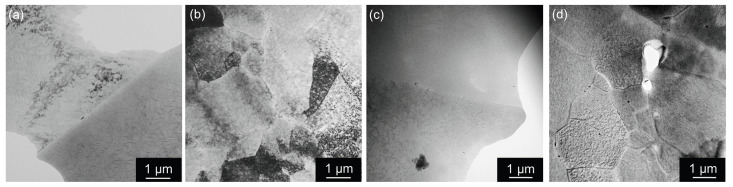
TEM micrographs of boundaries in materials after annealing at 300 °C/30 min, 450 °C/30 min, one CGP cycle performed at 300 °C, one solution treatment at 530 °C/30 min, 3% pre-straining and aging at 180 °C/14 h (near peak age condition), bright field: (**a**) AlLi MC, (**b**) AlLiSc MC, (**c**) AlLi TRC, and (**d**) AlLiSc TRC.

**Figure 21:**
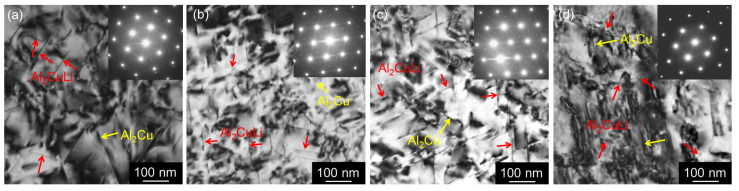
TEM micrograph of materials after annealing at 300 °C/30 min, 450 °C/30 min, one CGP cycle performed at 300 °C, one solution treatment at 530 °C/30 min, 3% pre-straining and aging at 180 °C/14 h (near peak age condition), zone [110]: (**a**) AlLi MC, (**b**) AlLiSc MC, (**c**) AlLi TRC, and (**d**) AlLiSc TRC.

Pre-straining before aging introduces additional dislocations in the crystal matrix, serving as nucleation sites for further precipitation (T8 temper). They almost entirely suppress the precipitation of (sub)grain boundary particles (see almost particles-free boundaries in all materials aged to near peak age condition at 180 °C/14 h in Figure 20). The precipitation of the $T_{1}$-Al_2_CuLi phase inside (sub)grains is enhanced to the detriment of the $\theta^{\prime }$-Al_2_Cu and $S^{\prime }$-Al_2_CuMg phases. Figure 21 shows bright field images taken in orientation [110] in pre-strained materials where the distribution of the $T_{1}$ phase becomes apparent with the corresponding feature of streaks in enclosed diffraction patterns.

## 4. Discussion

The significant differences in cooling rates between the experimental mold-casting and twin-roll casting and the presence of Sc produce vastly different microstructures in the as-cast state, which have lasting effects on post-processed materials. LOM images (Figure 1) confirm the grain-refining effect of Sc already in the as-cast state. A smaller grain size in both Sc-containing materials is evident. This grain refining effect of Sc on as-cast Al alloys is well characterized in the literature [57,58,59]. It is attributed to pre-formed ${\mathrm{Al}}_{3}\mathrm{Sc}$ particles that serve as effective heterogeneous nucleation sites due to the favorable crystal orientation relationship between α-Al and ${\mathrm{Al}}_{3}\mathrm{Sc}$ particles, modification of the microstructure of other refining particles (${\mathrm{Al}}_{3}\mathrm{Ti}$, ${\mathrm{Ti}}_{2}B$), or a formation of new complex phases containing Si and other trace or main alloying elements.

The primary phases are generally significantly coarser in the MC cast materials than in the TRC materials. They are formed at the boundaries of eutectic cells. However, the scale of these cells is significantly lower in the TRC materials (Figure 2). Generally, these differences are comparable to those observed in other Al-based systems [60]. The interdendritic spacings are between 50–100 µm in both MC materials, 20–30 µm in the TRC AlLi alloy, and 10–15 µm in the TRC AlLiSc alloy (Figure 2). Recent studies on ingot-cast Al-Li alloys [61,62] showed that homogenization time *t* and absolute homogenization temperature *T* are related by the homogenization kinetic equation [61]

(2) $$\frac{1}{T}=\frac{R}{Q}\ln\left[\frac{4\pi^{2}D_{0}t}{4.6L^{2}}\right],$$

where *L* is the interdendritic spacing, *Q* is the diffusion activation energy, *R* is the gas constant, and $D_{0}$ is a constant acting in the Arrhenius Equation (3) for diffusion coefficient *D* of the main alloying element with the smallest diffusivity

(3) $$D=D_{0}\exp\left(-\frac{Q}{RT}\right).$$

The homogenization time can be expressed by a reformulation of Equation (2)

(4) $$t=\frac{4.6L^{2}}{4\pi^{2}D_{0}}\exp\left[\frac{Q}{RT}\right].$$

By substitution of T=803.15K, $R=8.314\;J\cdot {\mathrm{mol}}^{-1}\cdot K^{-1}$, $Q\left(\mathrm{Cu}\right)=136.8\;\mathrm{kJ}\cdot {\mathrm{mol}}^{-1}$, and $D_{0}\left(\mathrm{Cu}\right)=0.084\;{\mathrm{cm}}^{2}\cdot s^{-1}$ into Equation (4), we obtain the values of the required homogenization time *t* (see Table 5).

The diffusion coefficient of Cu should be considered in our alloys because it is lower than the diffusion coefficients of Mg and Li [62]. The $D_{0}\left(\mathrm{Cu}\right)=0.084\;{\mathrm{cm}}^{2}/s$ and the Q(Cu)=136.8kJ/mol. Thus, the highest temperature, 530 °C (803 K), used during the thermal treatment of our alloys yields (Equation 4) annealing times of approximately 40–60 h for both MC materials and around 30 min for the TRC material. The selection of such a short annealing time is justified by EDS mapping confirming a full dissolution of Cu-bearing (Fe-free) primary phases in TRC AlLiSc alloy (Figure 9 and Figure 10). Our estimations clearly show the advantages of combining TRC and Sc microalloying, enabling the replacement of the energy and material-consuming homogenization with a simple homogenization/solution treatment just before quenching and the final age-hardening step.

Another consequence of this short exposure of the material to high temperatures is the suppression of an intensive Li depletion in the surface layer of the strip [63,64] and the elimination of the surface sculpting typical for ingot-cast and homogenized materials. This effect is often mentioned in the literature and generally detected through microhardness measurements in alloys with higher (2–3 wt.%) Li content [65,66]. On the contrary, microhardness maps of TRC strips (Figure 14c,d) do not exhibit the presence of any systematically softer surface layer.

The as-cast materials were annealed at 300 °C and 450 °C for 30 min each before the deformation by CGP. This two-step annealing should ensure the dissolution of low-melting point primary phases before the final solution step, and the formation of core-shell ${\mathrm{Al}}_{3}\left(\mathrm{Sc},\mathrm{Zr}\right)$ precipitates in Sc-containing alloys. This configuration is more stable and resistant to coarsening than binary ${\mathrm{Al}}_{3}\mathrm{Sc}$ precipitates, offering a more substantial grain refining effect than simple binary ${\mathrm{Al}}_{3}\mathrm{Zr}$ or ${\mathrm{Al}}_{3}\mathrm{Sc}$ precipitates [67,68]. Therefore, no Sc-containing material recrystallizes after solution treatment, and only pronounced fragmentation of grains into numerous subgrains occurs due to the intensive recovery (Figure 12).

The lack of dislocations serving as nucleation sites for strengthening particles during aging at 180 °C in solution-treated and quenched materials (T6 temper) and the presence of numerous subgrain boundaries in AlLiSc alloys result in the formation of particles with less pronounced strengthening effect: σ-Al_5_Cu_6_Li_2_ in the grain interior and coarse $S^{\prime }$-Al_2_CuMg preferentially on subgrain and grain boundaries (Figure 16, Figure 17, and Figure 21). This effect is typical for T6-treated materials [8,9,69] and generally should be suppressed by a small calibration pre-straining (3–10%) at room temperature before the age-hardening. This treatment (T8 temper) significantly accelerates the kinetics of age-hardening and increases the density of fine precipitates ($\theta^{\prime }$-${\mathrm{Al}}_{2}\mathrm{Cu}$, $T_{1}$-${\mathrm{Al}}_{2}\mathrm{CuLi}$) heterogeneously nucleating on dislocations [37,70,71,72]. Therefore, the peak-aged values are accessed at shorter annealing times (Figure 13). However, systematically persisting lower microhardness values (Figure 13b) and the presence of boundary precipitates in both AlLiSc materials (Figure 20b,d) indicate that larger pre-straining might entirely suppress the segregation of solutes on (sub)grain boundaries in this material. Recently, we analyzed the precipitate-strengthening effect for concentrations of alloying elements used in our alloy after the solution treatment of the as-cast state. Our estimations and similar assessments on more concentrated alloys show that the maximal microhardness increase could approach 80 HV0.1 in our alloy, providing further reserves for optimizing the processing strategy proposed in the present work [73,74,75].

## 5. Conclusions

A model near net shape procedure for manufacturing strips and thicker sheets for the aerospace industry from high-strength Al-Cu-Li-Mg-Zr alloys is proposed in this study. The novel strategy is based on appropriate post-processing of twin-roll cast alloy with a small addition of Sc. The key results could be summarized in the following manner:

1. Combining twin-roll casting of Al-Cu-Li-Mg-Zr and microalloying with a small amount of Sc has an essential impact on the size and distribution of primary intermetallic particles. The size of eutectic cells characterized by the interdendritic spacing is significantly reduced and, on average, does not exceed 10–15 μm.
2. Small dimensions of eutectic cells allow for the omission of energy-demanding long-term homogenization, generally coupled with a massive depletion of surface layers from Li atoms. Instead, a short multistep solution/homogenization treatment combined with a pre-deformation by the constrained groove pressing (300 °C/30 min, 450 °C/30 min, CGP, and 530 °C/30 min) could be used. A suitable distribution of small ${\mathrm{Al}}_{3}\left(\mathrm{Sc},\mathrm{Zr}\right)$ core-shell dispersoids stabilizing the fine-grained structure is achieved during this step.
3. Calibration pre-straining by 3% and final artificial aging at 180 °C/30 min assuring heterogeneous precipitation of a fine dispersion of reinforcing particles simulate the T8 temper typical for age-hardenable aluminum wrought alloys leading to optimal near peak-aged strengthening of the alloy.
4. The near net shape thickness of the strip allows for skip rolling or extruding, which are indispensable steps in conventionally cast materials. Both processes always produce strongly directional and anisotropic structures with flat and elongated (sub)grains prone to intergranular segregation, anisotropic corrosion, and intergranular delamination. The proposed procedure thus represents an optimal method for preparing lightweight high-strength materials from Al-Cu-Li-Mg-Zr alloy suitable for cryogenic applications in aeronautics.

## Figures and Tables

**Table 1:** Chemical composition of the studied materials in wt.%.

	Al	Cu	Li	Mg	Zr	Sc	Ag	Fe	Ti	V	Other
AlLi	95.98(9)	2.51(9)	0.73(6)	0.28(2)	0.12(6)	0.03(4)	0.24(8)	0.09(6)	0.01(1)	0.01(1)	<0.01
AlLiSc	95.79(9)	2.60(8)	0.71(8)	0.27(2)	0.11(7)	0.16(4)	0.24(7)	0.10(6)	0.01(1)	0.01(1)	<0.01

**Table 2:** Average interdendritic spacing *L* in as-cast materials evaluated by the mean linear intercept method.

	AlLi MC	AlLiSc MC	AlLi TRC	AlLiSc TRC
*L* [μm]	[135 ± 24]	[111 ± 22]	[12 ± 2]	[13 ± 3]

**Table 3:** EDS point analysis corresponding to Figure 3—chemicalcomposition in at.%.

Spot	Note	Al	Cu	Mg	Fe	Sc
1	Sc-rich	(78 ± 5)	(14.7 ± 0.6)	(1.4 ± 0.2)	(0.9 ± 0.2)	(5.0 ± 0.3)
2	Sc-rich	(93 ± 5)	(1.1 ± 0.8)	(1.2 ± 0.5)	(0.5 ± 0.4)	(4.2 ± 0.8)
3	Cu-rich	(75 ± 5)	(22.1 ± 0.6)	(2.0 ± 0.3)	(0.8 ± 0.1)	(0.1 ± 0.1)
4	Cu-rich	(75 ± 5)	(20.8 ± 0.8)	(2.0 ± 0.3)	(1.1 ± 0.2)	(1.1 ± 0.2)
5	Mg-rich	(92 ± 4)	(4.0 ± 0.3)	(3.6 ± 0.3)	(0.2 ± 0.1)	(0.2 ± 0.1)
6	Mg-rich	(85 ± 5)	(8.1 ± 0.5)	(6.3 ± 0.6)	(0.4 ± 0.2)	(0.2 ± 0.1)
7	matrix	(98 ± 3)	(0.6 ± 0.0)	(1.1 ± 0.1)	(0.0 ± 0.0)	(0.3 ± 0.1)
8	Fe-Cu-rich	(74 ± 5)	(15.5 ± 0.7)	(1.3 ± 0.3)	(9.0 ± 0.4)	(0.2 ± 0.1)

**Table 4:** Average grain diameter *d* in solution-treated materials calculated by the mean linear intercept method from Figure 12 via Equation (1).

	AlLi MC	AlLiSc MC	AlLi TRC	AlLiSc TRC
$d_{CGP+sol.}$ [μm]	[98 ± 3]	[54 ± 5]	[92 ± 15]	[24 ± 3]

**Table 5:** Homogenization times according to Equation (4) and interdendritic spacing displayed in Table 2.

	AlLi MC	AlLiSc MC	AlLi TRC	AlLiSc TRC
Homogenization time *t*	56 h	37 h	27 min	32 min

## Data Availability

The original contributions presented in this study are included in the article. Further inquiries can be directed to the corresponding author.
